# First Genomic Analysis of Dendritic Cells from Lung and Draining Lymph Nodes in Murine Asthma

**DOI:** 10.1155/2015/638032

**Published:** 2015-02-24

**Authors:** Thomas Tschernig, Christina Hartwig, Andreas Jeron, Quoc Thai Dinh, Marcus Gereke, Dunja Bruder

**Affiliations:** ^1^Institute of Anatomy and Cell Biology, Saarland University, Campus Homburg, Kirrberger Straße 100, 66424 Homburg, Germany; ^2^Institute of Pharmacology, Medical School of Hannover, Carl-Neuberg-Straße 1, 30625 Hannover, Germany; ^3^Infection Immunology Group, Institute of Medical Microbiology, Infection Control and Prevention, Otto-von-Guericke University Magdeburg, Leipziger Straße 44, 39120 Magdeburg, Germany; ^4^Department of Experimental Pneumology, Saarland University, Campus Homburg, Kirrberger Straße 100, 66424 Homburg, Germany; ^5^Immune Regulation Group, Helmholtz Centre for Infection Research, Inhoffenstraße 7, 38124 Braunschweig, Germany

## Abstract

Asthma is the consequence of allergic inflammation in the lung compartments and lung-draining lymph nodes. Dendritic cells initiate and promote T cell response and drive it to immunity or allergy. However, their modes of action during asthma are poorly understood. In this study, an allergic inflammation with ovalbumin was induced in 38 mice versus 42 control animals. After ovalbumin aerosol challenge, conventional dendritic cells (CD11c/MHCII/CD8) were isolated from the lungs and the draining lymph nodes by means of magnetic cell sorting followed by fluorescence-activated cell sorting. A comparative transcriptional analysis was performed using gene arrays. In general, many transcripts are up- and downregulated in the CD8^−^ dendritic cells of the allergic inflamed lung tissue, whereas few genes are regulated in CD8^+^ dendritic cells. The dendritic cells of the lymph nodes also showed minor transcriptional changes. The data support the relevance of the CD8^−^ conventional dendritic cells but do not exclude distinct functions of the small population of CD8^+^ dendritic cells, such as cross presentation of external antigen. So far, this is the first approach performing gene arrays in dendritic cells obtained from lung tissue and lung-draining lymph nodes of asthmatic-like mice.

## 1. Introduction

Dendritic cells play a key role not only in asthma during the initiation of the allergic immune response but also in the effector phase of the allergic inflammation leading to typical clinical symptoms [1, 2]. Allergy pathophysiology hereby reveals both similarities and clear differences between humans and mice. Basically, the dendritic cells can be divided into three groups: a small population of plasmacytoid dendritic cells, a predominant population of conventional dendritic cells, and, during inflammation, the monocyte-derived or inflammatory dendritic cells [3]. The dendritic cells isolated and analysed in this study were the so-called conventional dendritic cells, which are positive for CD11c and MHCII [4]. In addition, the expression of CD8 was used to separate CD8^+^ from CD8^−^ dendritic cells. Upon comparison, fewer CD8^+^ dendritic cells than CD8^−^ ones were found in the lung tissue. The CD8^+^ dendritic cells were more concentrated in the draining lymph nodes, making them a lymph node-resident dendritic cell population [4, 5]. Furthermore, within lymph nodes the CD8^+^ dendritic cells contribute to cytotoxic T cell responses via cross presentation of exogenous antigens [2, 4, 6]. CD8^−^, but not the CD8^+^, sorted dendritic cells from schistosoma-infected mice prevented allergic responses [7]. CD8^+^ and CD8^−^ dendritic cells from BCG-infected mice suppressed allergic T cell responses* in vitro* and* in vivo* [8].

In recent years, the expression of CD103 and CD11b has been introduced for phenotyping dendritic cells in asthma and elsewhere. The lymphoid resident dendritic cells are characterized as CD103^−^ dendritic cells (CD11b^+^, CD8^+^, and CD8^−^). In contrast the nonlymphoid residents are characterized as CD103^+^ dendritic cells (CD11b^+^, CD8^+^, and CD8^−^) [3]. Our approach to the gene expression of conventional dendritic cells compared CD8^−^ and CD8^+^ conventional dendritic cells, revealing an interesting panel of regulated genes. Since there is a close relation between dendritic cells in the tissue and the draining lymph nodes, both compartments were taken for analysis. The majority of dendritic cells pick up allergen not only in the bronchi but also in the alveoli and migrate to lymph nodes where the allergen is presented to B cell and T cells initiating and maintaining humoral and cellular lymphocyte responses. Lymphocytes become activated and recirculate through the tissues including the lung where dendritic cell immigration and activation are mediated [1, 2].

## 2. Aims

The present study had the aim to compare the gene expression of distinct dendritic cells isolated from the lung tissue and the lung-draining lymph nodes in mice with induced asthmatic-like inflammation and controls. A further aim of the presented study was to compare lung tissue and lymph node-derived dendritic cells from control animals and animals suffering from allergic inflammation. Obtaining enough cell numbers of dendritic cell subsets for gene expression analysis is challenging. The more the subsets that are defined using multiple markers, the more the difficult the harvesting of a sufficient number of dendritic cells. Therefore, a strategy was chosen to obtain sufficient numbers of dendritic cells in a medium scale approach, using less than fifty animals each for the disease group and the control group. The classical combination of CD11c and MHCII defined the small numbers of conventional dendritic cells which yielded the draining mediastinal lymph nodes. For the bigger lung tissue yield of dendritic cells, the expression of CD8*α*, which is relevant for pulmonary allergy, was additionally included. Data from gene arrays in murine macrophages and dendritic cells from lung tissue is available [9], but to our knowledge there is no data on gene arrays in dendritic cells from allergic or asthmatic-like inflamed lungs. Furthermore, no approach could be found including the analysis of dendritic cells from lung-draining lymph nodes.

## 3. Materials and Methods

The sensitization and allergen challenge of mice were performed as described before [4]. All experiments were carried out using C57BL/6 mice (8–12 weeks old, Charles River, Sulzfeld, Germany). Mice (*n* = 38) were sensitized by intraperitoneal injection of 10 *μ*g ovalbumin (Grade VI) emulsified in 1.5 mg aluminium hydroxide in a total volume of 150 *μ*L on days 1, 14, and 21. Control mice (*n* = 42) were sham-sensitized with 1.5 mg alum in PBS. OVA provocation (1% OVA Grad V in PBS for 20 min) was performed on days 28 and 29 on all mice. Lungs were obtained and cut into small fragments, digested with collagenase, and DNAse and enriched by gradient centrifugation and magnetic depletion of granulocytes, lymphocytes, and erythrocytes. Bronchial lymph nodes cells were isolated by passing the tissue through a metal mesh, directly followed by the magnetic depletion of granulocytes, lymphocytes, and erythrocytes. Remaining cells isolated from lung tissue and bronchial lymph nodes were resuspended in PBS and stained for 30 minutes with *α*-CD11c, *α*-MHCII, and *α*-CD8 (BD Biosciences). After washing, the stained cells were analysed and DCs (CD11c^high^ and MHCII^high^) were sorted by using a Mo-Flo-System (Cytomation). The total RNA was isolated using the RNeasy Mini Kit (Qiagen, Hilden, Germany) according to the manufacturer's recommendations. Samples for microarray analysis were generated by applying an mRNA-specific double linear amplification protocol (Affymetrix). Briefly, double-stranded cDNA was generated* in vitro* in a reverse transcription using the T7dT23 primer (5′-GGCCAGTGAATTGTAATACGACTCACTATAGGGAGGCGG(T)23-3′; Metabion, Planegg, Germany) and SuperScript II reverse transcriptase (Invitrogen, Karlsruhe, Germany), followed by a second-strand cDNA synthesis involving DNA polymerase I (Invitrogen) and* E. coli* DNA ligase (Invitrogen). For the first amplification round the Promega P1300 RiboMax Kit for T7 amplification (Promega, Mannheim, Germany) was used to synthesize unlabeled cRNA from the purified cDNA. A second amplification round was performed starting with the amplified cRNA and reverse transcription by using random hexamer primers (Pharmacia, Freiburg, Germany) and SuperScript II reverse transcriptase for the first-strand synthesis. The second-strand synthesis, again using T7dT23 primers and additional RNase H treatment, was performed as mentioned above. For the final amplification round, the GeneChip expression 3′-Amplification Reagent Kit for labeling (Affymetrix, San Francisco, CA, USA), producing biotinylated cRNA, was used. The quantity and quality of biotinylated cRNA were checked photometrically. Samples were fragmented and hybridized to a Mouse Genome 430 2.0 Array (Affymetrix). Washing and staining were performed as recommended by the manufacturer. Analysis of microarray data was performed using GeneSpring GX 10.0 software (Agilent Technologies). The robust multiarray analysis (RMA) algorithm was used for normalization.

## 4. Results and Discussion

The experiments and sorting procedures were performed with animal groups of 5 to 7 animals and repeated to finally reach the number of 42 control mice and 38 asthmatic-like mice for the cell isolation from lung tissue and the number of 15 control and 15 asthmatic-like mice for the cell isolation from lung-draining lymph nodes. The cells were deep-frozen and pooled for analysis in the gene arrays. The yield of dendritic cells from lung tissue was as follows. From 42 control mice in total 121.000 CD8^+^ and 843.000 CD8^−^ dendritic cells (CD11c^+^/MHCII^+^) were obtained from the tissue of whole lungs. From 38 asthmatic-like mice in total 118.000 CD8^+^ and 740.000 CD8^−^ dendritic cells were obtained again from the lung tissue. The yield of dendritic cells from the lung-draining lymph nodes was as follows. From 15 control mice in total 168.000 dendritic cells (CD11c^+^/MHCII^+^) were obtained and from 15 asthmatic-like mice 140.000 dendritic cells were obtained. The reanalysis in flow cytometry led to a purity of at least 95%, and in most experiments, the purity was 97 to 99%.

**Figure 1:**
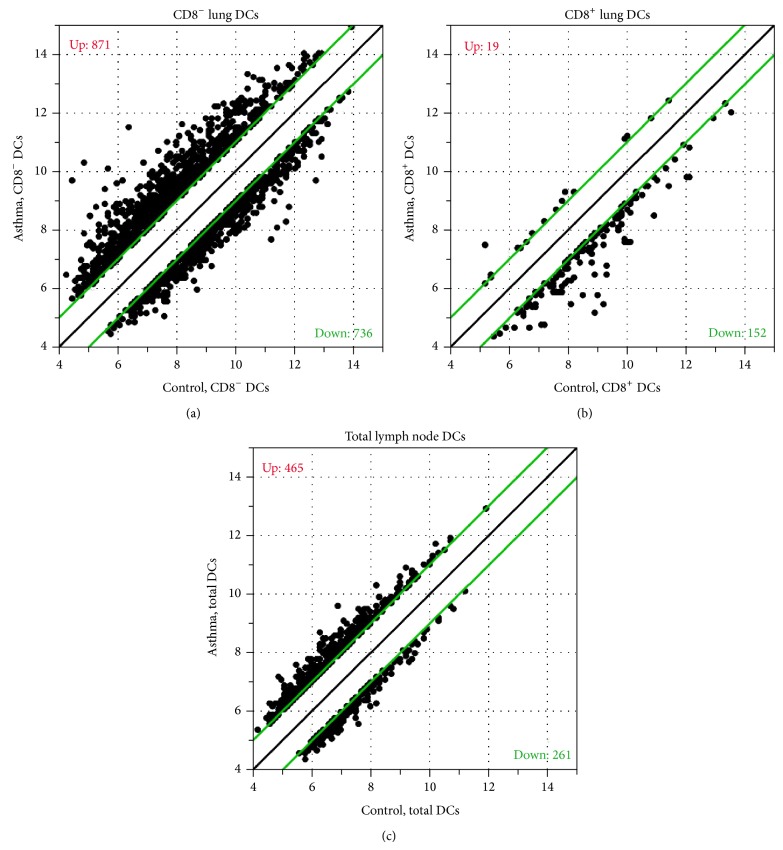
Scatterplots of DC microarrays. Normalized signal intensities of microarrays from the depicted DC subsets derived from lungs (a) and (b) and lung-draining lymph nodes (c) of asthma and control mice are plotted against each other. Only transcripts with a fold change of > ±2 (indicated by green lines) in the according comparison are shown. The numbers of up- and downregulated transcripts are depicted as well.

In the CD8^−^ subset of lung tissue-derived dendritic cells 871 transcripts were upregulated with a fold change > ±2 in the comparison between induced allergic inflammation and controls (Figure 1). Again a high number, 736, of transcripts were downregulated. This is in contrast to the CD8^+^ subset in the same comparison, where only 19 transcripts were upregulated and 152 were downregulated (Figure 1). The CD8^+^ subpopulation is more constant and the few genes regulated are downregulated (Figure 1). This does not exclude a distinct function of the CD8^+^ DC subpopulation, like cross presentation of external antigen, for instance.

Since considerable lower numbers of dendritic cells could be obtained from the lung-draining lymph nodes, all dendritic cells were analyzed and again those from asthmatic-like lungs were compared to those of control lungs. Here, 465 transcripts were upregulated and 261 were downregulated. One can speculate whether the CD8^−^ dendritic cells, being part of the sorted lymph node DCs were again those with the most regulated transcripts. It is known that, during inflammation, there is a constant flow of migrating dendritic cells to the draining lymph nodes, which induces massive changes in the cellular microenvironment in the lymph node [2]. It can be assumed that the majority of those migratory dendritic cells were CD8^−^. But the transcriptional changes in the lymph nodes are moderate. This is interesting and may support a role of the lymph nodes in controlling the immune response and inflammation.

**Figure 2:**
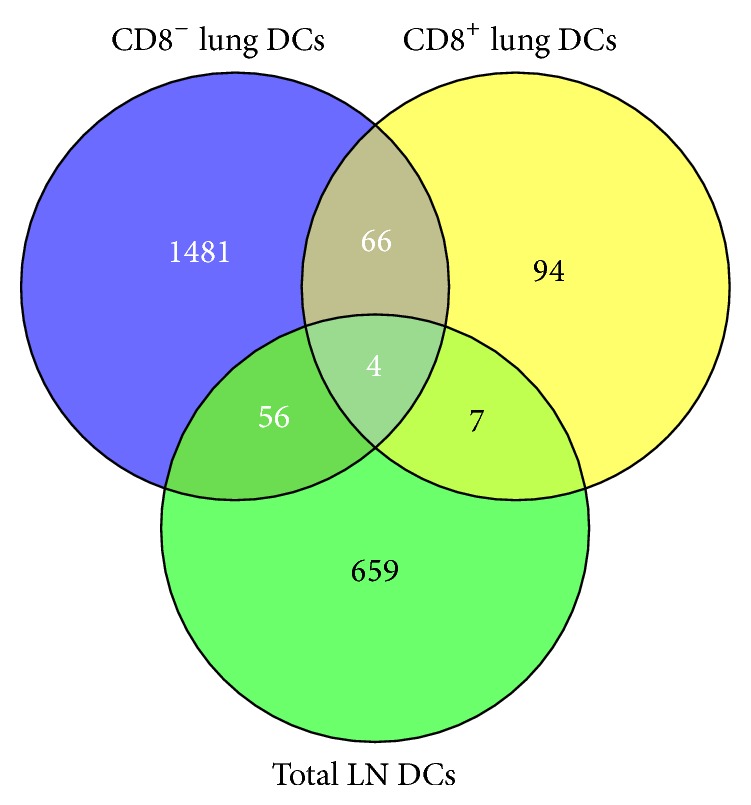
Venn diagram of regulated transcripts. The numbers and overlap of regulated transcripts (>±2-fold) in the depicted DC subset from lungs and lung-draining lymph nodes derived from asthma versus control animals are shown, respectively.

A Venn diagram (Figure 2) was used to display the overlap between regulated transcripts in all three populations: 66 were shared between the CD8^−^ and CD8^+^ dendritic cell population and 4 were shared by all dendritic cells, including the lymph node-derived ones. The small overlap of regulated genes amongst the three DC populations may underline their phenotypical distinctness. Moreover, the fact that the total lymph node DCs contain both CD8^+^ and CD8^−^ DC populations, but still show little overlap between the regulated genes found in asthmatic-lung-derived CD8^+^ or CD8^−^ DCs clearly shows that DC functions in asthma are dependent on the cellular environment. On the assumption that many CD8^−^ dendritic cells had migrated to the lymph nodes, the intense interaction with the stimulated micromilieus might have changed their phenotype.

In Tables 1 and 2, gene enrichment analysis of CD8^−^ dendritic cells is presented because CD8^−^ dendritic cells seemed to be the most highly regulated during allergic inflammation. In the KEGG pathway enrichment significantly regulated genes have been found, which are involved in chemokine signaling, cytokine/receptor-interaction, Fc*γ*R-mediated phagocytosis, and TLR signaling (Table 1). No significant KEGG pathway enrichment was found in the comparison of CD8^+^ dendritic cells (asthma versus control, see Supplemental Table  5 in Supplementary Materials available online at http://dx.doi.org/10.1155/2015/638032).

In the GO analysis, mainly changes of the plasma membrane and vesicles are prominent (Table 2). In the CD8^+^ DC GO analysis, changes of the plasma membrane were prominent (Supplemental Table  5).

In the dendritic cells from draining lymph nodes consisting of total dendritic cells (CD8^−^ and CD8^+^), only few components are overrepresented (Supplemental Table  6). No significant changes occurred on cell surfaces but rather intracellular compartments (Table 3) seem to be modulated which might be related to the intense antigen processing and antigen presentation which is the core task of dendritic cells in the lymph node.

From the abundant regulated genes in the CD8^−^ dendritic cells highly upregulated genes were, amongst many others, distinct serpins,* Arl5b*, and* Kif3b*  (supplemental material, excel sheet of regulated genes). Since it was not the primary aim of this study, no candidate genes were selected to perform confirming PCR analyses.

## 5. Asthma-Induced Differential Gene Expression in CD8^−^ Lung DCs

The most prominent transcriptional alterations could be observed in the CD8^−^ DC compartment from asthmatic-like mice when compared to CD8^−^ DC from control mice. In general genes that are being upregulated more than 10-fold under these conditions are, for example,* Ear11*,* Abcd2*,* CD209e*,* Fabp1*, and* Slc7a2*.* Ear11*, officially known as* Rnase2a*, encodes for the “Ribonuclease, RNase A family, 2A (liver, eosinophil-derived neurotoxin)” protein. It is +34.7-fold upregulated in asthmatic-like conditions. It has been previously reported to be asthma-induced in short-term, intermediate term, and long-term ovalbumin exposure of the lung [10].* Abcd2* is upregulated +13.6-fold. It encodes for the “ATP-binding cassette, subfamily D (ALD), member 2,” a protein belonging to the large group of ATP-binding cassette (ABC) transporters responsible for cross membrane transport of various substances.* Abcd2* is, for example, involved in the peroxisomal import of fatty acids.* CD209e* also known as* Signr4* is a mouse homologue to the human DC-SIGN protein. DC-SIGN is a type II C-type lectin that functions as an adhesion molecule on the surface of dendritic cells. In the context of asthma, it has been reported that DC-SIGN on human MDDCs mediates cellular responses to, for example, Bermuda grass pollen antigens* in vitro* leading to the production of TNF-*α* [11].* Fabp1* (fatty acid binding protein 1, liver) is upregulated +10.5-fold in CD8^−^ DCs from asthmatic-like mice. That fatty acid binding proteins in principle are involved in DC function that has been demonstrated with regard to* Fabp4* (also known as* aP2*), another protein from the same family with at least nine members [12]. Mice lacking* aP2* were shown to produce less IL-12 and TNF and were less potent in inducing T cell proliferation [13]. However, expression of* Fabp1* on DCs in an asthmatic-like context has not been described so far.* Slc7a2* stands for “solute carrier family 7 (cationic amino acid transporter, y+ system), member 2” and belongs to the amino acid-polyamine-organocation family of transport proteins.* Slc7a2* is involved in the cellular uptake of arginine, lysine, and ornithine.

A comparably small group of genes in CD8^−^ DCs were found to be downregulated in an asthma-dependent manner. For example, the two heat shock proteins 1a and 1b (*Hspa1a* and* Hspa1b*) show a drastic loss of expression (−10.6- and 10.3-fold).* Hspa1a* and* Hspa1b* are also known as heat-shock 70-kD proteins 1a and 1b, respectively. They are involved in cellular stress responses and act as chaperons. Of note, the toll-like receptor 3 (*Tlr3*) was also found to be downregulated −4.6-fold.* Tlr3* is a pattern recognition receptor sensing double-stranded RNA typically associated with viral infections.

As the cell surface is an important immunological interface of DCs, it is interesting to observe that genes whose protein products are associated with the cell surface are significantly enriched in the gene ontology analysis. In this category, genes like* Htr2c*,* CD2*,* CD200r4*, and* CD22* may be of interest. The serotonin receptor 2c (*Htr2c*) is highly upregulated (+7.1-fold) under asthmatic-like conditions.* Htr2c* expression has been also demonstrated on epidermal DCs in context of a contact allergy model in mice [14]. The T lymphocyte surface antigen* CD2* is +5.1-fold upregulated. Interestingly, CD2^+^ human pDCs were shown to comprise a distinct DC population producing higher amounts of IL-12p40 and expressing higher levels of costimulatory CD80 compared to CD2^−^ pDCs following an influenza A virus infection of the lung [15]. The CD200 receptor 4 (*CD200r4*) is upregulated +4.6-fold on CD8^−^ DCs under allergic conditions and is a receptor for the Ox-2 ligand (also known as CD200). The CD200/CD200r-axis is thought to regulate myeloid cell activity. This is of particular interest since it has been recently reported in a rat asthma model that local delivery of recombinant CD200 strongly reduces OVA-induced lung accumulation of myeloid DCs in the lung [16].* CD22* also known as* Siglec2* is upregulated 4-fold in CD8^−^ DCs and belongs to the family of sialic-acid-binding lectins. Originally CD22 is thought to be a B cell restricted protein inhibiting the B-cell antigen receptor (BCR) signaling [17]. However, there are reports demonstrating its expression also on pDCs [18].

On the other hand, the induction of asthma led in CD8^−^ DCs also to the reduced expression of some surface proteins or at least their according transcripts. The killer cell lectin-like receptors* Klrb1b* (−5.6-fold),* Klrd1* (−4.0-fold, also known as* CD94*) and* Klrk1* (−2.1-fold, also known as* Nkg2d*) are all downregulated in CD8^−^ DCs compared to nonasthmatic-like conditions.

Interestingly, genes involved in antigen presentation like* H2-Oa* and* H2-Ob* which encodes for the histocompatibility 2 O region loci alpha and beta are slightly downregulated (*H2-Oa*: −2.7-fold,* H2-Ob*: −3.2-fold). Of note, the costimulatory protein* CD86*, working in conjunction with MHC class II proteins to activate CD4^+^ T cells, is downregulated as well (−2.2-fold).

With regard to the lysosomal compartment genes like* Pla2g15*,* Sort1*, and several cathepsins are regulated in CD8^−^ DCs from asthmatic-like mice. Phospholipase A2 Group 15 (*Pla2g15*, +5.3-fold) is involved in the eicosanoid synthesis, a substance class also containing prostaglandins and leukotrienes which have pro- and anti-inflammatory potential.

Sortilin 1 (*Sort1*, +4.7-fold) very efficiently binds serum lipoproteins but can act as a multiligand type-1 receptor. The group of cathepsins (cathepsins A, B, D, F, K, and L) is upregulated in CD8^−^ DCs from asthmatic-like lungs (*Ctsa*: +3.2-fold,* Ctsb*: +2.1-fold,* Ctsd*: +3.0-fold,* Ctsf*: +3.3-fold,* Ctsk*: +2.4-fold, and* Ctsl*: +3.3-fold). Cathepsins represent a group of endoproteases typically abundant in the lysosomal compartment and hydrolytically degrade, for example, the extracellular matrix and basal membranes.

In the functional group of “chemokine and cytokine signaling,” genes like* CD24* (+6.1-fold),* Ccl24* (+6.1-fold), and* Pdgfc* (+5.8-fold) were highly upregulated.* CD24* is upregulated +6.1-fold. CD24 is a protein that is able to provide costimulatory signals to T cells and has been described to occur in the context of DC differentiation from CD8*α* ^−^ to CD8*α* ^+^ DCs [19]. Interestingly, it was reported that CD24-deficient mice exhibit increased susceptibility to danger but not pathogen-associated molecular patterns [20].* Ccl24* is a chemokine also known as eotaxin-2 and is upregulated +6.1-fold in CD8^−^ DCs. Ccl24 is a ligand for Ccr3 and is able to recruit eosinophils [21]. Platelet-derived growth factor C polypeptide (*Pdgfc*, +5.8-fold) is a potent mitogen for cells of mesenchymal origin [22]. Furthermore, genes like* Lepr* (leptin receptor; +4.7-fold),* Adcy3* (adenylate cyclase 3; +3.7-fold),* Ccr1* (chemokine C-C motif receptor 1; +2.7-fold),* Ccl6* (chemokine C-C motif ligand 6; +2.4-fold),* Ccl8* (chemokine C-C motif ligand 6; +2.4-fold), and* Cxcr3* (chemokine C-X-C motif receptor 3; −4.5-fold) were differentially regulated to a smaller extend.

## 6. Conclusions

Dendritic cells and their subsets play a key role in initiating and maintaining allergic inflammation. Dendritic cells are present in low numbers in lung tissue and in very low numbers in lung-draining lymph nodes. Gene analysis requires the separation of dendritic cells from huge numbers of animals, limiting the analysis of very rare subsets. The present analysis showed a regulation, up and down, of many more transcripts in the CD8^−^ conventional dendritic cells of the lung tissue as compared to the CD8^+^ DCs supporting the pathophysiological predominance of the CD8^−^ subset. Surprisingly, the transcriptional reaction in the dendritic cells of the draining lymph nodes was moderate indicating the role of the lymph nodes more as a stabilizer or controller than as a booster of the allergic inflammation. Conventional CD8^−^ and CD8^+^ dendritic cells are distinct subsets with differentiated roles in allergic inflammation. Further investigations will investigate whether the sorting of alternative dendritic cell subsets will show an overlap to those analyzed in this study.

## Supplementary Material

Supplemental table 1: List of 1607 differentially regulated genes in CD8- lung DCs.
Supplemental table 2: List of 171 differentially regulated genes in CD8+ lung DCs.
Supplemental table 3: List of 726 differentially regulated genes in total lymph node DCs.
Supplemental table 4: KEGG pathway and gene ontology analysis of differentially regulated genes in CD8- lung DCs.
Supplemental table 5: KEGG pathway and gene ontology analysis of differentially regulated genes in CD8+ lung DCs.
Supplemental table 6: KEGG pathway and gene ontology analysis of differentially regulated genes in total lymph node DCs.

## Figures and Tables

**Table 1:** KEGG pathway enrichment of 1607 regulated transcripts in CD8^−^ lung DCs.

KEGG pathway	KEGG pathway ID	Total number of genes in pathway	Number of genes found	% genes found	Bonferroni corrected term-enrichment *P* value
Lysosome	4142	123	29	23.6	1.7*E* − 07
Chemokine signaling pathway	4062	185	29	15.7	1.7*E* − 03
Peroxisome	4146	79	17	21.5	1.8*E* − 03
PPAR signaling pathway	3320	81	17	21.0	2.5*E* − 03
Cytokine-cytokine receptor interaction	4060	248	34	13.7	5.7*E* − 03
Fc gamma R-mediated phagocytosis	4666	92	17	18.5	1.4*E* − 02
Toll-like receptor signaling pathway	4620	101	18	17.8	1.4*E* − 02
Hematopoietic cell lineage	4640	84	16	19.0	1.5*E* − 02
B cell receptor signaling pathway	4662	78	15	19.2	2.2*E* − 02
Phagosome	4145	177	25	14.1	3.6*E* − 02
Focal adhesion	4510	199	27	13.6	4.1*E* − 02

Transcripts that are found to be differentially regulated in CD8^−^ lung DCs from asthmatic-like versus healthy control mice were analyzed for statistical overrepresentation in KEGG pathway annotations using a one-sided hypergeometric test and Bonferroni *P* value correction. Only KEGG pathways with a *P* value <0.05 were considered to be overrepresented.

**Table 2:** Gene ontology analysis of 1607 regulated transcripts in CD8^−^ lung DCs.

Cellular component	GO ID	Total number of genes in category	Number of genes found	% genes found	Bonferroni corrected term-enrichment *P* value
Cytoplasm	5737	2435	201	8.3	1.7*E* − 07
External side of plasma membrane	9897	145	29	20.0	2.4*E* − 07
Cell surface	9986	202	34	16.8	1.1*E* − 06
Plasma membrane	5886	1127	104	9.2	4.9*E* − 05
Plasma membrane part	44459	783	77	9.8	2.0*E* − 04
Cytoplasmic part	44444	1706	141	8.3	2.7*E* − 04
Lytic vacuole	323	57	11	19.3	2.8*E* − 02
Lysosome	5764	57	11	19.3	2.8*E* − 02
Basolateral plasma membrane	16323	79	13	16.5	4.4*E* − 02

Transcripts that are found to be differentially regulated in CD8^−^ lung DCs from asthmatic-like versus healthy control mice were analyzed for statistical overrepresentation in “cellular component” GO annotations using a one-sided hypergeometric test and Bonferroni *P* value correction. Only GO terms with a *P* value <0.05 were considered to be overrepresented.

**Table 3:** Gene ontology analysis of transcripts regulated in total DCs from lung-draining lymph nodes.

Cellular component	GO ID	Total number of genes in category	Number of genes found	% genes found	Bonferroni corrected term-enrichment *P* value
Intracellular	5622	5869	239	4.1	5.0*E* − 11
Intracellular part	44424	5799	236	4.1	1.0*E* − 10
Intracellular organelle	43229	4906	205	4.2	2.2*E* − 09
Intracellular membrane-bounded organelle	43231	4389	174	4.0	2.4*E* − 05
Intracellular organelle part	44446	2247	103	4.6	4.3*E* − 05
Cytoplasm	5737	4078	162	4.0	8.2*E* − 05
Nucleus	5634	2346	104	4.4	1.8*E* − 04
Cytoplasmic part	44444	3065	127	4.1	3.2*E* − 04
Intracellular non-membrane-bounded organelle	43232	1127	58	5.1	8.6*E* − 04
Z disc	30018	54	8	14.8	1.2*E* − 02
I band	31674	61	8	13.1	2.8*E* − 02
Cell cortex	5938	99	10	10.1	4.6*E* − 02

Transcripts that are found to be differentially regulated in total lymph node DCs from asthmatic-like versus healthy control mice were analyzed for statistical overrepresentation in “cellular component” GO annotations using a one-sided hypergeometric test and Bonferroni *P* value correction. Only GO terms with a *P* value <0.05 were considered to be overrepresented.
