# Supplementary material for: First Genomic Analysis of Dendritic Cells from Lung and Draining Lymph Nodes in Murine Asthma
Source: Int J Genomics. 2015 Feb 24;2015:638032. doi: 10.1155/2015/638032 (PMC4355561; doi:10.1155/2015/638032)
Supplement: Supplementary file 1 — Supplemental table 1: List of 1607 differentially regulated genes in CD8- lung DCs. Supplemental table 2: List of 171 differentially regulated genes in CD8+ lung DCs. Supplemental table 3: List of 726 differentially regulated genes in total lymph node DCs. Supplemental table 4: KEGG pathway and gene ontology analysis of differentially regulated genes in CD8- lung DCs. Supplemental table 5: KEGG pathway and gene ontology analysis of differentially regulated genes in CD8+ lung DCs. Supplemental table 6: KEGG pathway and gene ontology analysis of differentially regulated genes in total lymph node DCs. [file 638032.f1.zip › 638032.f1/mat.638032.v1.pdf]

KEGG Pathway	KEGG pathway ID	Total number of genes in category	Number of genes found	% genes found	Bonferroni corrected term-enrichment p value
no significant enrichment	-	-	-	-	-

Cellular component	GO ID	Total number of genes in category	Number of genes found	% genes found	Bonferroni corrected term-enrichment p value
intracellular	5622	5869	239	4.1	5.0E-11
intracellular part	44424	5799	236	4.1	1.0E-10
intracellular organelle	43229	4906	205	4.2	2.2E-09
intracellular membrane-bounded organelle	43231	4389	174	4.0	2.4E-05
intracellular organelle part	44446	2247	103	4.6	4.3E-05
cytoplasm	5737	4078	162	4.0	8.2E-05
nucleus	5634	2346	104	4.4	1.8E-04
cytoplasmic part	44444	3065	127	4.1	3.2E-04
intracellular non-membrane-bounded organelle	43232	1127	58	5.1	8.6E-04
Z disc	30018	54	8	14.8	1.2E-02
I band	31674	61	8	13.1	2.8E-02
cell cortex	5938	99	10	10.1	4.6E-02

Biological process	GO ID	Total number of genes in category	Number of genes found	% genes found	Bonferroni corrected term-enrichment p value
negative regulation of biological process	48519	1930	102	5.3	1.7E-06
negative regulation of cellular process	48523	1760	91	5.2	4.4E-05
cellular macromolecule metabolic process	44260	3017	131	4.3	9.3E-04
regulation of metabolic process	19222	2469	112	4.5	9.3E-04

regulation of macromolecule metabolic process	60255	2113	99	4.7	1.1E-03
regulation of developmental process	50793	1017	57	5.6	1.6E-03
positive regulation of biological process	48518	2158	100	4.6	1.6E-03
regulation of cell differentiation	45595	718	42	5.8	1.3E-02
regulation of primary metabolic process	80090	2137	95	4.4	1.7E-02
protein metabolic process	19538	1846	84	4.6	2.4E-02
cellular protein metabolic process	44267	1565	73	4.7	3.9E-02
cellular protein modification process	6464	1353	65	4.8	4.4E-02
protein modification process	36211	1353	65	4.8	4.4E-02

**Moecular function**	**GO ID**	**Total number of genes in category**	**Number of genes found**	**% genes found**	**Bonferroni corrected term-enrichment p value**
no significant enrichment	-	-	-	-	-
